# Pullout strength of pedicle screws with cement augmentation in severe osteoporosis: A comparative study between cannulated screws with cement injection and solid screws with cement pre-filling

**DOI:** 10.1186/1471-2474-12-33

**Published:** 2011-02-01

**Authors:** Lih-Huei Chen, Ching-Lung Tai, De-Mei Lee, Po-Liang Lai, Yen-Chen Lee, Chi-Chien Niu, Wen-Jer Chen

**Affiliations:** 1 Department of Orthopaedic Surgery, Chang Gung Memorial Hospital, Chang Gung University, Taoyuan, Taiwan; 2 Graduate Institute of Medical Mechatronics, Department of Mechanical Engineering, Chang Gung University, Taoyuan, Taiwan

## Abstract

**Background:**

Pedicle screws with PMMA cement augmentation have been shown to significantly improve the fixation strength in a severely osteoporotic spine. However, the efficacy of screw fixation for different cement augmentation techniques, namely solid screws with retrograde cement pre-filling versus cannulated screws with cement injection through perforation, remains unknown. This study aimed to determine the difference in pullout strength between conical and cylindrical screws based on the aforementioned cement augmentation techniques. The potential loss of fixation upon partial screw removal after screw insertion was also examined.

**Method:**

The Taguchi method with an L_8 _array was employed to determine the significance of design factors. Conical and cylindrical pedicle screws with solid or cannulated designs were installed using two different screw augmentation techniques: solid screws with retrograde cement pre-filling and cannulated screws with cement injection through perforation. Uniform synthetic bones (test block) simulating severe osteoporosis were used to provide a platform for each screw design and cement augmentation technique. Pedicle screws at full insertion and after a 360-degree back-out from full insertion were then tested for axial pullout failure using a mechanical testing machine.

**Results:**

The results revealed the following 1) Regardless of the screw outer geometry (conical or cylindrical), solid screws with retrograde cement pre-filling exhibited significantly higher pullout strength than did cannulated screws with cement injection through perforation (*p *= 0.0129 for conical screws; *p *= 0.005 for cylindrical screws). 2) For a given cement augmentation technique (screws without cement augmentation, cannulated screws with cement injection or solid screws with cement pre-filling), no significant difference in pullout strength was found between conical and cylindrical screws (*p >*0.05). 3) Cement infiltration into the open cell of the test block led to the formation of a cement/bone composite structure. Observations of the failed specimens indicated that failure occurred at the composite/bone interface, whereas the composite remained well bonded to the screws. This result implies that the screw/composite interfacial strength was much higher than the composite/bone interfacial strength. 4) The back-out of the screw by 360 degrees from full insertion did not decrease the pullout strength in any of the studied cases. 5) Generally, larger standard deviations were found for the screw back-out cases, implying that the results of full insertion cases are more repeatable than those of the back-out cases.

**Conclusions:**

Solid screws with retrograde cement pre-filling offer improved initial fixation strength when compared to that of cannulated screws with cement injection through perforation for both the conically and cylindrically shaped screw. Our results also suggest that the fixation screws can be backed out by 360 degrees for intra-operative adjustment without the loss of fixation strength.

## Background

Osteoporosis is a common disease in aging populations. Spinal surgeons unavoidably encounter patients with osteoporosis who need spinal decompression and instrumentation due to degenerative spinal diseases. However, pedicle screw instrumentation in a severely osteoporotic spine remains a challenge for orthopedic surgeons.

Previous studies have demonstrated that the holding power of screw in non-augmented osteoporotic bone decreases with decreasing bone mineral density [1-3]. Consequently, to date, efforts to improve screw holding power have focused primarily on the pullout of screws augmented with polymethylmethacrylate (PMMA) [4-8], calcium phosphate [9-11] and calcium sulfate [12,13] in osteoporotic bone.

PMMA augmentation is regarded as an efficient method to enhance screw strength in osteoporotic bones [4-8]. Traditionally, to improve the anchoring strength of screws in osteoporotic bone, PMMA is injected directly into the prepared pilot hole of the vertebral body prior to screw insertion. The pedicle screw is then inserted into the cement to enhance the screw anchoring strength. Another insertion technique involves the usage of an expandable screw, which allows for flange expansion at the screw tip and, hence, increases the screw holding power [14,15]. Recently, works have focused on the perforated screw with PMMA augmentation, which allows for the injection of cement through the perforation to achieve the improvement of screw anchoring strength [4,16,17]. Although numerous studies address the improvement in pullout strength with various screw augmentation techniques, a comparison of screw insertion technique between solid screws with retrograde cement pre-filling and cannulated screws with cement injection through perforation is lacking.

Our review of the literature found that most of the current research on pedicle screw pullout has been performed with cylindrically shaped pedicle screws [4,5,7,18-20]. Cross sections of pedicles at the lumbar spine have an elliptical shape. In the anterior-posterior direction, the pedicle margin converges [21]. Conical screws were developed to better match the anatomical situation in the pedicle. Conically shaped screws were demonstrated to provide better fixation strength when compared to cylindrically shaped screws [22,23]. Theoretically, conical screws should progressively compress the surrounding bone with each turn of the screw during insertion. This compression of the surrounding bone should be beneficial, as it provides increased purchase during screw insertion [24,25]. However, as a result of the rapid reduction in the compression of the surrounding bone if the screw is partially removed to adjust the screw placement during surgery, the reduction of the compression stress could also cause a rapid reduction in the fixation strength. The effect of partial screw removal on the pullout strength is a concern for surgeons because it is often necessary to adjust the insertion depth during screw placement.

Studies have investigated the mechanical performance of pedicle screws in vertebrae, but only minimal data are available concerning the performance of conical screws in the presence of compromising events such as partial screw removal. In the present study, we address two features of the pullout strength of conical versus cylindrical screws: 1) the fixation strength between solid screws with retrograde cement pre-filling and cannulated screws with cement injection through perforation and 2) the effect of partial screw removal after full insertion.

## Methods

### Taguchi factorial design

The Taguchi method with an L_8 _array was employed to determine the significance of design factors. Three factors were considered in evaluating the holding power of a fixation screw inserted into synthetic bone. The three factors were screw shape (conical/cylindrical), cement augmentation technique (solid screws with cement pre-filling/cannulated screws with cement injection) and screw insertion type (full insertion/back-out). Each factor was further assigned into two levels. Therefore, a total number of 8 trials (2^3^) were required to identify the relative significance of design factors using a full factorial approach. Table 1 lists the selected factors and definitions of their corresponding levels.

**Table 1:** Parameters selection and levels definition

Design factor	Level 1	Level 2
Screw shape	Conical	Cylindrical
Cement augmentation technique	PMMA pre-filling (Solid screw)	PMMA injection (Cannulated screw)
Screw insertion type	Full insertion	Back-out

### Synthetic bone samples

Synthetic bone (model #1522-505, Pacific Research Laboratory Inc., Vashon Island, WA, USA) made from polyurethane foam was used as substitute for cadaveric spinal bone because of its consistent and homogeneous structural properties. The synthetic bone was supplied as rectangular shape (test block) with the dimensions of 13 cm × 18 cm × 4 cm; the material was open-cell rigid polyurethane foam with a density of 0.09 g/cm^3^, which simulates a cadaveric spinal bone with extreme osteoporosis [26-29].

### Bone screws

**Figure 1:**
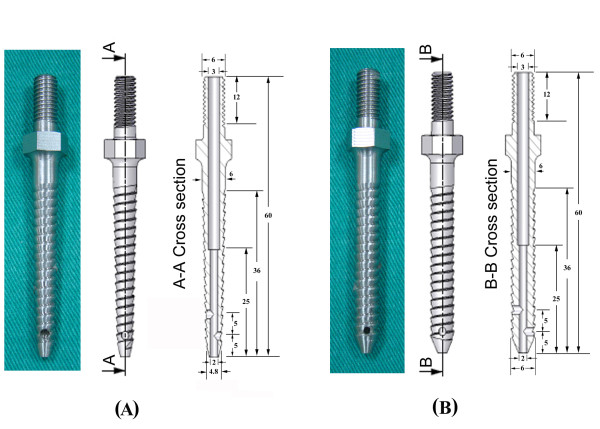
**A photo and diagram of the cannulated screws**. (A) Conical and (B) cylindrical screws. The outer geometry of the solid screws is identical to that of the cannulated screws but without the internal cavity. (Dimension: mm).

Four screw designs were employed in the present study: conical-solid, conical-cannulated, cylindrical-solid and cylindrical-cannulated. The outer geometry of conical and cylindrical screws differed mainly in the taper of their major and minor diameters from the hub to the screw tip. The cylindrical screws maintained a constant diameter from hub to tip; in contrast, the conical screws tapered 20%, from 6.0 mm at the hub (major diameter) to 4.8 mm at the tip. For both screw types, the thread pitch was 2 mm and the thread depth was 0.8 mm. The thread contour, a proprietary characteristic, was identical for both screws. For the cannulated screws, two radial holes with a diameter of 2 mm were located at 5-mm increments along the length of screw starting at the screw tip. Two sets of outer threads were made, one with a length of 32 mm from the screw tip and another with a length of 12 mm from the screw head. The outer thread on the screw head was fixed in a cylindrical rod with a matched inner thread in the subsequent pullout test. The conical and cylindrical screws in the cannulated design are illustrated in Figure 1.

### Allocation of the specimens

The allocation of specimens to experimental groups is shown in Table 2. The following ten different combinations of screw designs and augmentation techniques were tested (six replicates in each group):

**Table 2:** Allocation of the Specimens to Experimental Groups

Group	Screw Shape	Solid/Cannulated	Augmentation	Full Insertion/Back-Out	Specimen Number
1	Conical	Solid	Retrograde PMMA pre	Full Insertion	6
2	Conical	Solid	Retrograde PMMA pre	Back-Out	6
3	Conical	Solid	None	Full Insertion	6
4	Conical	Cannulated	PMMA injection through perforation	Full Insertion	6
5	Conical	Cannulated	PMMA injection through perforation	Back-Out	6

6	Cylindrical	Solid	Retrograde PMMA pre	Full Insertion	6
7	Cylindrical	Solid	Retrograde PMMA pre	Back-Out	6
8	Cylindrical	Solid	None	Full Insertion	6
9	Cylindrical	Cannulated	PMMA injection through perforation	Full Insertion	6
10	Cylindrical	Cannulated	PMMA injection through perforation	Back-Out	6

Group 1: Conical-solid screws, retrograde cement pre-filling and full screw insertion.

Group 2: Conical-solid screws, retrograde cement pre-filling and a 360-degree screw back-out after full insertion.

Group 3: Conical-solid screws with screw full insertion and no cement augmentation.

Group 4: Conical-cannulated screws, cement injection through perforation and full screw insertion.

Group 5: Conical-cannulated screws and cement injection through perforation with a 360-degree screw back-out after full insertion.

Group 6: Cylindrical-solid screws, retrograde cement pre-filling and full screw insertion.

Group 7: Cylindrical-solid screws, retrograde cement pre-filling and a 360-degree screw back-out after full insertion.

Group 8: Cylindrical-solid screws with full screw insertion and no cement augmentation.

Group 9: Cylindrical-cannulated screws, cement injection through perforation and a full screw insertion.

Group 10: Cylindrical-cannulated screw, cement injection through perforation and a 360-degree screw back-out after full insertion.

### Specimen preparation

For cannulated screws (both conical and cylindrical), PMMA cement was injected into the test block after screw insertion. A pilot hole was drilled into the test block using a 3-mm drill bit, and a cannulated screw was then inserted into the test block through the prepared pilot hole. The insertion rate for all screws was 3 rev/min [30]; a countdown timer was used to measure the screw insertion rate. All screws were inserted to identical depths using a consistent depth gauge, and radiological examinations were performed to check the implanted screw depths. Following the cannulated screw insertion, Osteobond bone cement (Zimmer, Warsaw, IN) was mixed at room temperature and introduced into the cannulated screws using a self-designed cement injector system that exerts pressure on the cement. The cement injector was composed of a cement gun, syringe, adapter and cannulated screw. One minute after the cement powder and monomer were mixed, the liquid-phase cement was transferred into a 10-ml syringe, which was then inserted into the cement gun. An adapter was used to connect the syringe to the cannulated screw. For all specimens, a total of 3 ml of cement was injected into the cannulated screw. The insertion technique for the cannulated screw was identical to that described in our previous work [4]

For solid screws (both conical and cylindrical), the solid screw was inserted into the test block through the prepared pilot hole and then removed to create a hole with identical dimension as the screw contour (conical or cylindrical). A total of 3 ml of cement was then retrogradely injected into the created hole using a 4-mm diameter bone biopsy needle (Allegiance, Healthcare Co., McGaw Park, Illinois, USA). A mark was made with aseptic marking pen on the needle. The length from the marking point to the needle tip was 5 mm shorter than the length of selected screw. Next, the biopsy needle was inserted into the prepared pilot hole until the marking point approached the entry edge of the test block. Cement was then injected into the pilot hole accompanied by progressive needle retraction out of the test block, until a total volume of 3 ml of bone cement was injected. With this technique, a uniform distribution of cement can be achieved. Following the pre-filling of bone cement, the solid screw was then fully inserted into the test block. To evaluate the effect of partial screw removal, the screws were randomly rotated out by 360 degrees from full insertion four minutes after the introduction of PMMA cement.

### Biomechanical tests

**Figure 2:**
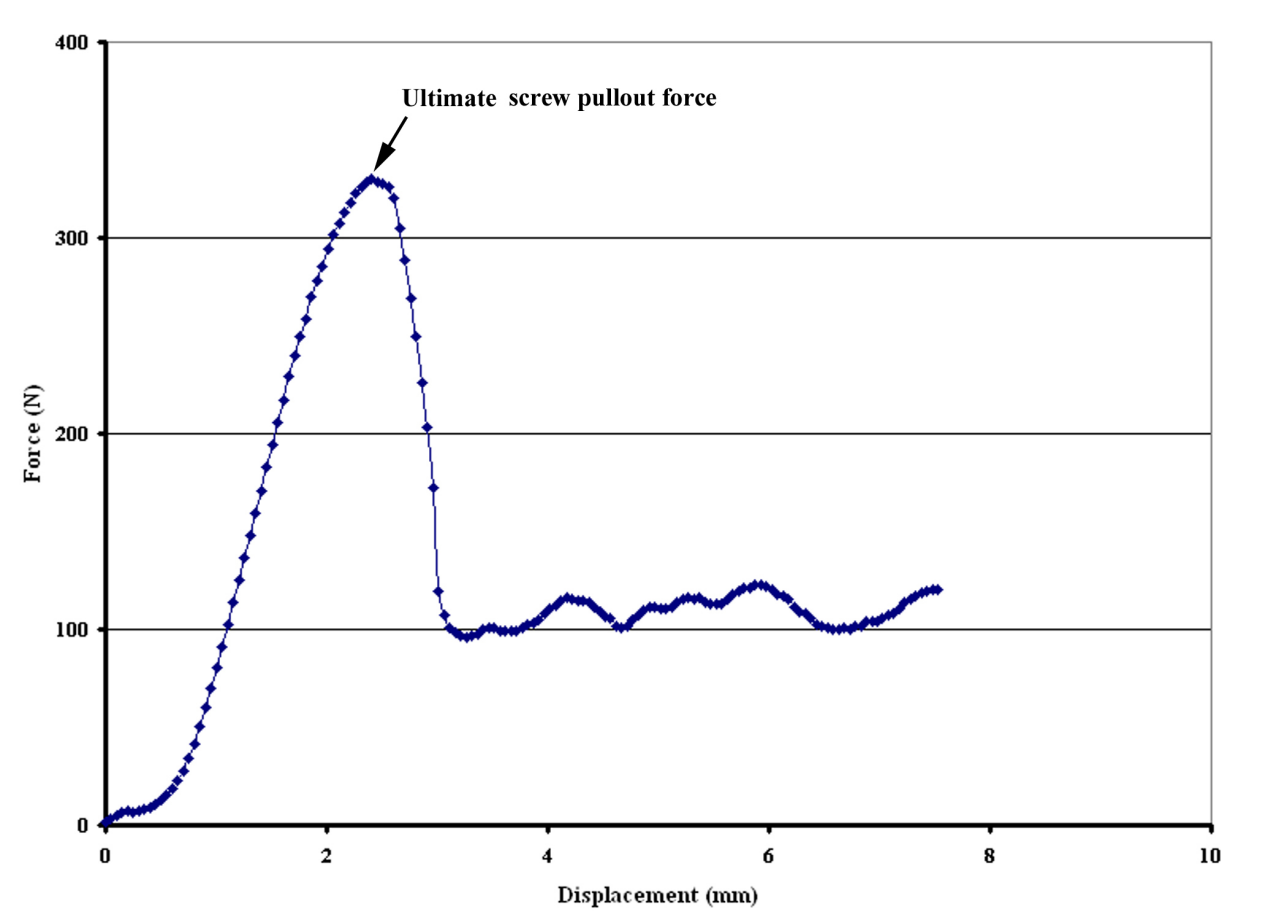
**An example of a force-displacement failure curve for a cylindrical cannulated screw**. The ultimate pullout strength was defined as the maximum load before the curve slope first becomes negative.

The method for screw pullout test was identical to that used in our previous study [4]. The individual specimen was tested for failure in axial pullout using an Instron testing machine (model 5544, Instron Inc., Canton, MA, USA). The test block, with a screw inserted, was placed on a specially designed universal fixture with a self-aligning function to ensure vertical pullout alignment. The pedicle screw was attached to the testing machine by a rod threaded to the head of the screw. After the specimens were mounted, pullout force was applied at a constant crosshead rate of 5 mm/min [30]. The force acting on the screw during testing was continuously recorded in 0.1 mm increments (sampling rate: 0.83 Hz) until the peak pullout resistance was reached, displacing the screw outwards. The peak force recorded during the pullout test was defined as the maximum pullout strength for comparison. Six trials for each screw fixation configuration were performed, and the mean value for the maximum pullout strength of the six trials was determined. An example of this force-displacement curve is shown in Figure 2.

### Statistical analysis

To evaluate the effects of screw designs (conical or cylindrical) and different modes of screw implantation (cannulated screw with cement injection or solid screw with retrograde cement pre-filling) on the stability of spinal fixation, the magnitudes of the ultimate pullout force were statistically compared. Unpaired two-tailed Student's *t*-tests were performed for the intergroup comparison. Differences were considered significant at *p *< 0.05.

## Results

**Figure 3:**
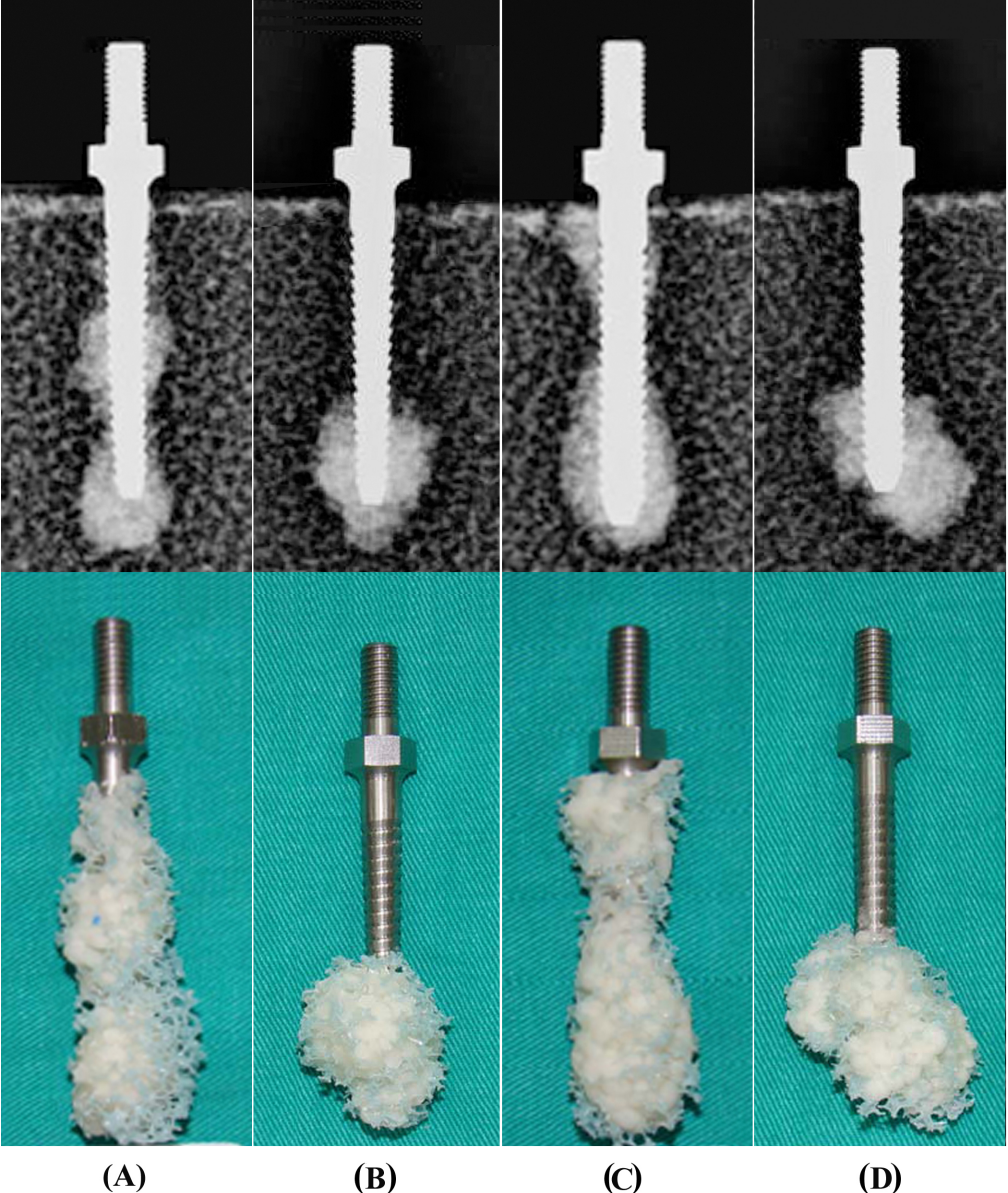
**A radiological photograph showing the test block and the inserted conical and cylindrical screws following cement augmentation (top) and specimens after the pullout tests (bottom)**. (A) A conical solid screw with cement pre-filling, (B) a cylindrical solid screw with cement pre-filling, (C) a conical cannulated screw with cement injection and (D) a cylindrical cannulated screw with cement injection.

The radiological and physical examinations of the screws inserted into the test blocks and specimens after the pullout tests are shown in Figure 3. The radiological photographs (Figure 3, top) indicated that the area of the cement/screw interface was greater for solid screws with retrograde cement pre-filling than for cannulated screws with cement injection. Observations of the failed specimens after the pullout test (Figure 3, bottom) indicated that cement infiltration into the open cell of the test block led to formation of a (cement/bone) composite structure. All of the failures occurred at the composite/bone interface; however, the composite remained well bonded to the screws, implying that the screw/composite interfacial strength was much higher than the composite/bone interfacial strength.

**Figure 4:**
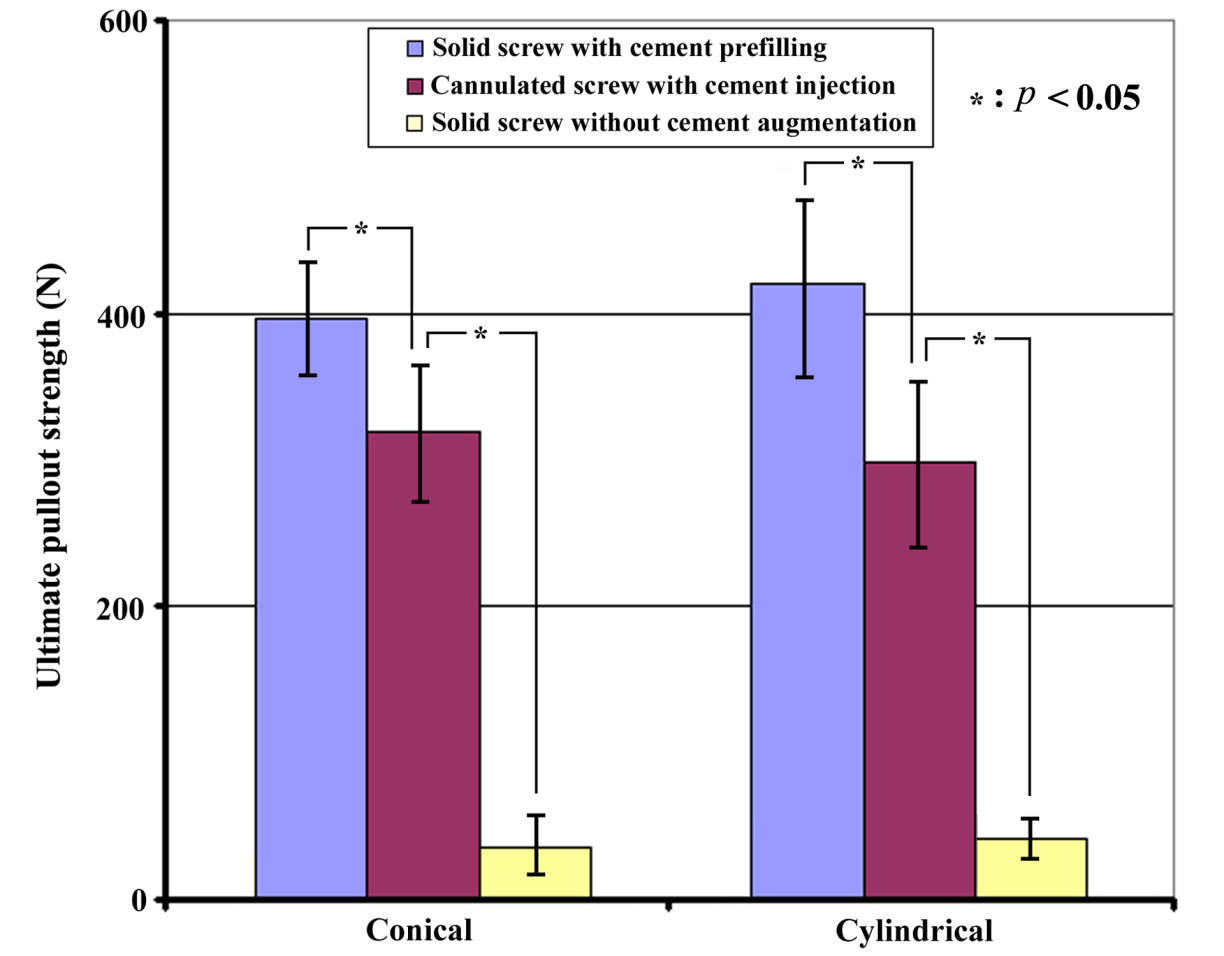
**The average ultimate pullout strength of fully inserted conical and cylindrical pedicle screws with various screw fixation techniques**. Regardless of the screw outer geometry (conical or cylindrical), solid screws with retrograde cement pre-filling exhibited the highest pullout strength, whereas solid screws without cement augmentation exhibited the lowest pullout strength (*p <*0.001).

The average ultimate pullout strengths of fully inserted conical and cylindrical pedicle screws for the different cement augmentation techniques are shown in Table 3 and Figure 4. Regardless of the screw outer geometry (conical or cylindrical), solid screws with retrograde cement pre-filling exhibited significantly higher pullout strength than that of the cannulated screw with retrograde cement pre-filling (*p *= 0.0129 for conical screws; *p *= 0.005 for cylindrical screws); whereas solid screws without cement augmentation exhibited the lowest pullout strength (*p <*0.001). For the conical screws, the solid type provided a 23% increase in the pullout strength when compared to the cannulated type (*p *= 0.0129). In contrast, for the cylindrical screws, the solid type provided a 41% increase in the pullout strength when compared to the cannulated type (*p *= 0.005). For a given screw augmentation technique (screw without cement augmentation, cannulated screw with cement injection or solid screw with cement pre-filling), no significant difference in pullout strength was found between conical and cylindrical screws (*p *> 0.05).

**Table 3:** Ultimate pullout strength of fully inserted conical and cylindrical pedicle screws with various screw insertion techniques. (Unit: Newton)

	Conical Screw			Cylindrical Screw		
		
Specimen number	Solid Screw with PMMA prefilling	Cannulated Screw with PMMA injection	Solid Screw without PMMA augmentation	Solid Screw with PMMA prefilling	Cannulated Screw with PMMA injection	Solid Screw without PMMA augmentation
1	346	298	47	357	240	45
2	365	355	22	372	249	34
3	442	308	54	469	348	62
4	416	245	18	371	246	27
5	376	351	15	463	340	37
6	426	368	52	493	364	49
	
Ave.	396	321	35	421	298	42
SD	37	46	18	60	59	13

P-value:

*p *= 0.0129 (Solid Screw with PMMA prefilling vs. Cannulated Screw with PMMA injection, Conical Screw)

*p *= 0.005 (Solid Screw with PMMA prefilling vs. Cannulated Screw with PMMA injection, Cylindrical Screw)

**Figure 5:**
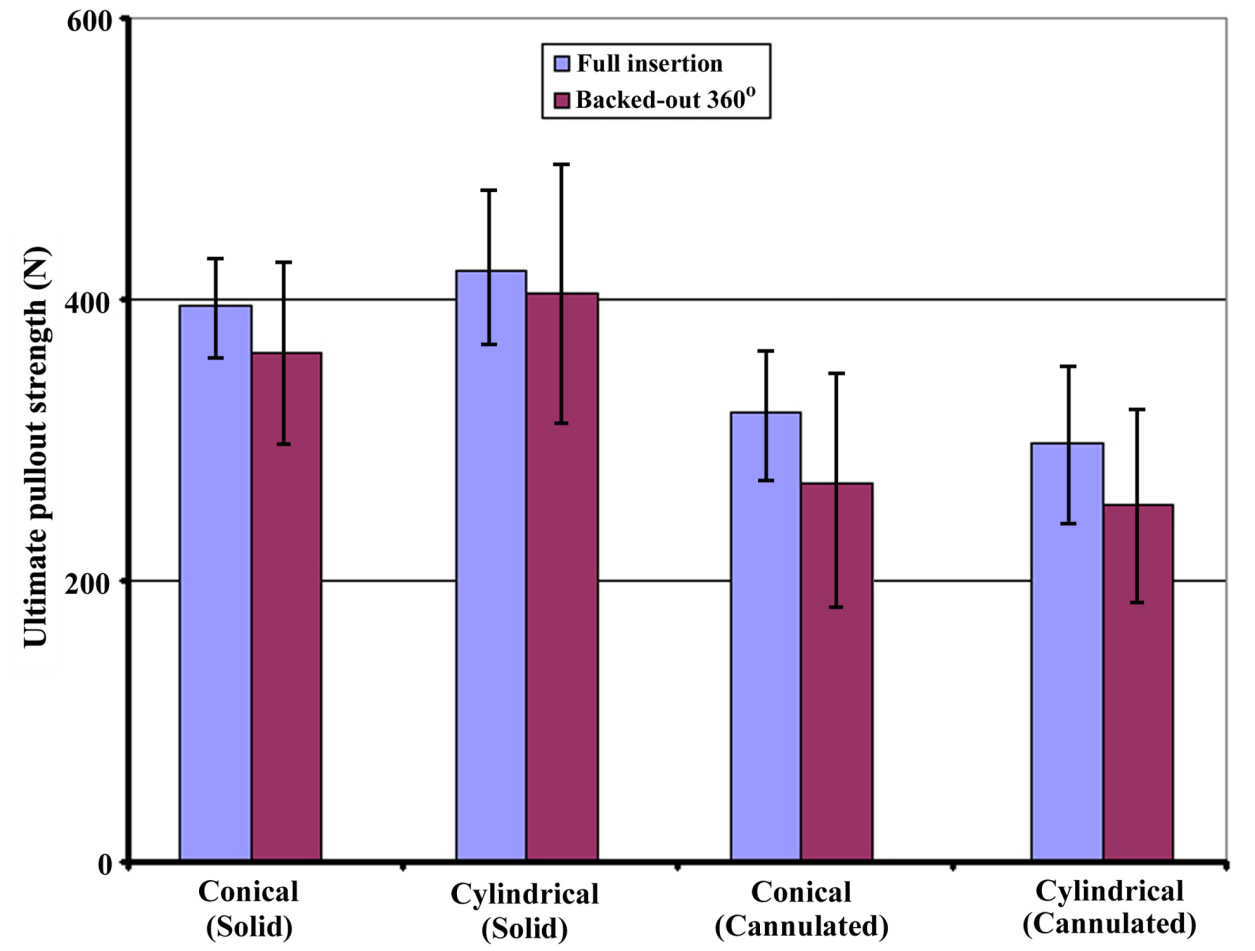
**The average ultimate pullout strength for conical and cylindrical pedicle screws tested at full insertion and after a 360-degree screw back-out**. The pullout strengths were unchanged after partial removal (not significant).

The average ultimate pullout strength for conical and cylindrical pedicle screws tested at full insertion and after a 360-degree screw backout is shown in Figure 5. Regardless of the screw outer geometry (conical or cylindrical), the pullout strengths were unchanged (not significant) after the partial removal from full insertion. Additionally, the pullout strength for pedicle screws after partial removal had a larger standard deviation than that after full insertion.

The Taguchi analysis indicated that the design factor "cement augmentation technique" was the main influential factor, whereas "screw shape" was the least influential factor affecting the pullout strength.

## Discussion

Adequate pedicle screw fixation in the presence of compromised bone quality presents a challenging problem for spine surgeons. Cancellous bone is more profoundly affected by osteoporosis process than cortical bone, and it is not surprising that the anchoring strength is significantly decreased in patients with low bone mineral density.

Cancellous bone is generally reported to have a density in the range of 0.09 to 1.25 g/cm^3 ^[26]. The variations in apparent density, trabeculae orientation and mechanical properties of cancellous bone within and between specimens are large. Consequently, a very large number of tests are required to isolate the effects of screw design. The use of synthetic cancellous bone simplifies the experimental set-up, thus limiting experimental error. In the present study, commercially available artificial osteoporotic bones with an open celled structure (test blocks) were used as a substitute for human osteoporotic vertebrae. The manufacturer's instruction states the following: The appearance of open cell rigid foam resembles that of human cancellous bone [28]. The test blocks offer a uniform and consistent density that eliminates the variability encountered when testing with human cadaver bones [31]. Consequently, test blocks are suitable for a variety of applications requiring an open-cell structure, such as cement injection and the modeling of osteoporotic cancellous bone. Many biomechanical studies using polyurethane foam (test block) to simulate osteoporotic cancellous bone have shown that polyurethane foam is a good alternative for in vitro testing [3,27,29,32]. In recent studies, Hashemi et al. [32] compared the axial pullout strength and insertion torque of augmented and nonaugmented pedicle screws using polyurethane foam with densities of 0.16 and 0.32 g/cm^3^, corresponding to the porosity of osteoporotic and normal bones, respectively. Their results revealed a significant correlation between peak pullout resistance and insertion torque. Zehnder et al. [29] investigated the effects of screw orientation and load to failure of a plate/bone construct that was attached to 0.09 g/cm^3 ^polyurethane foam (used to simulate severely osteoporotic cancellous bone). They concluded that in a severely osteoporotic model, failure in cantilever bending at low forces would take place regardless of fixation methods used and the added benefit of oblique screw placement observed in healthy bone is not observed in osteoporotic bone. In addition, Ramaswamy et al. [3] compared the pullout strengths of four different commercially available cannulated screws inserted in polyurethane foam blocks with three different densities (0.16, 0.24 and 0.32 g/cm^3^) simulating osteoporotic, osteopenic and normal bones, respectively. Their results indicated that the holding power of screws is directly correlated to bone density, thread design and number of threads engaging the bone. Reinsertion through the same hole could reduce the ultimate pullout strength. Additionally, Patel et al. [27] performed the compressive test on different densities (0.32, 0.16 and 0.09 g/cm^3^) of polyurethane foams to examine whether the commercially polyurethane foams are suitable for mimicking human osteoporotic cancellous bone. The fracture stresses of these foams enable them to be used as models for normal (0.32 g/cm^3^), osteoporotic (0.16 g/cm^3^) and very low density osteoporotic cancellous bone (0.09 g/cm^3^). They concluded that the 0.16 g/cm^3 ^polyurethane foam is a good alternative for in vitro testing because it has compressive Young's modulus and yield strength values similar to osteoporotic bone that has also been tested in compression. In the current study, in considering of the ease of cement injection, the polyurethane foam with a density of 0.09 g/cm^3 ^was chosen for emphasis of extremely osteoporotic cancellous bone. We believe that although the pullout test did not measure the actual screw/bone interfacial strength, the 0.09 g/cm^3 ^polyurethane foam provides a uniform platform to compare the mechanical behavior of pedicle screws with various designs.

Few reports have addressed the effects of partial screw removal on the bone/screw interfacial strength [22,33]. However, the reported results have been inconsistent. Abshire et al. [22] investigated the characteristics of pullout failure in conical and cylindrical pedicle screws after full insertion and partial removal using porcine lumbar vertebrae. They concluded that there was no reduction in the pullout strength, stiffness or work to failure when pedicle screws were partially removed either by 180 or 360 degrees, from full insertion. Lill et al. [33] examined the mechanical performance of cylindrical and dual-core pedicle screws that were fully inserted and then removed 4 mm from calf and human vertebrae. Their results indicated that partially removing the screws before cyclic loading led to an increase in displacement of 32%, which resulted in a significant reduction in the screw pullout strength. In the current study, we report that back-out of the screw by 360 degrees did not reduce the pullout strength; our results were inconsistent with those of Lill et al. Reasons causing the insistent results might be attributed to the fact that in their investigation into the effect of screw removal in calf and human vertebrae, PMMA cement was not used and severe osteoporosis was not considered. Another factor causing the inconsistent results might be the absence of the cortex shell in the current study. Nevertheless, our results indicate that in a synthetic material with a density similar to that of severely osteoporotic bone, screw back-out does not affect the pullout strength of screws with cement augmentation.

PMMA cement infiltration into the open cell of the test block led to the formation of a composite cement/bone structure in the area of cement infiltration (Figure 3). For all of the cases, observation of the failed specimens revealed that the failure occurred at the composite/bone interface, whereas the composite remained well bonded to the screws (Figure 3, bottom). In the present study, for a given screw design (conical or cylindrical), we report a significantly higher pullout strength for solid screws with retrograde cement pre-filling than for cannulated screws with cement injection using artificial osteoporotic bones (Figure 4). Our results demonstrate that solid screws with retrograde cement pre-filling offer improved initial screw fixation strength in severe spinal osteoporosis. The radiological examinations indicated that the area of the composite/bone interface was greater for the solid screws with retrograde cement pre-filling than for the cannulated screws with cement injection (Figure 3). This increased interface led to significantly higher pullout strength for solid screws. For the cannulated screws, the distal placement of the radial holes provided a longer distance for the cement to flow outside from the distal end of screw. This would result in most cement being distributed on the distal end of the screw and would reduce the area of the composite/bone interface enormously. In contrast to the cannulated screw, cement is prefilled prior to screw insertion for solid screws. During screw insertion, the prefilled cement is squeezed to occupy some of the voids of the adjacent trabecular bone, which distributes cement on the more proximal threads of the solid screw because of a host of factors such as cement wettability, porosity considerations, flow rate, etc. This causes a difference in density between the bone infiltrated with cement and the adjacent intact trabecular bone. The enormous difference in density between composite structure and intact bone was thought to induce a general failure mode along the composite/bone interface during axial screw pullout. Theoretically, the pullout force required to remove the composite structure (bone with cement infiltration) from adjacent intact trabecular bone is proportional to the composite/bone contact area. Consequently, we believe that a greater area of the composite/bone interface will be beneficial to the anchoring strength of the screw. Although cement pre-filling prior to screw insertion benefits the pullout strength, cement leakage from the screw insertion point is more likely with cement pre-filling (Figure 3). This result implies that the use of solid screws with retrograde cement pre-filling technique has an associated risk of cement leakage into the spinal canal.

The Taguchi method utilizing an orthogonal array has proven to be a useful tool to markedly reduce the total number of required experiments. The method contains a well-chosen subset of all possible test condition combinations and can achieve a balanced comparison of levels of any factor [34-36]. In the current study, although all factorial trials had been performed, a further investigation using the Taguchi method to determine the main contribution of the design factors was appropriate. The Taguchi results indicated that, rather than eight trials, only four trials were required to precisely determine the most influential factors. The Taguchi's L_8 _array analysis revealed the design factor "cement augmentation technique" was the main influential factor, whereas "screw shape" was the least influential factor affecting the pullout strength. In addition, the highest pullout strength was found in combination of cylindrical-solid-full insertion. The results of Taguchi analysis were consistent with our conclusion that the PMMA augmentation technique for solid screws with retrograde cement pre-filling offers improved initial fixation strength.

Our study is an in vitro analysis of specimens prepared in a laboratory environment, which does not necessarily represent clinical circumstances. There are limitations to this study. First, a test block was used as a substitute for human osteoporotic vertebrae. Although the synthetic bone provides a platform for comparison of pullout strength, the material properties of the test block are somewhat different from those of actual osteoporotic cancellous bone; the extrapolation of our results to clinical utilization should be performed with caution. Second, the measurement of pullout strength at the screw-bone interface did not take into account the cortical shell of the spinal vertebrae, which may have an impact on interfacial bonding strength. However, our specimens were all prepared and tested in a uniform, reproducible manner, and we believe that our results provide a comparison of the mechanical performance of various screws in severely osteoporotic bone. Third, the volume of injected cement tested was constant (3 ml). The amount of injected cement might be an important influential factor in determining the screw holding power. The effects of the amount of injected cement on bone/screw interfacial strength deserved to be conducted in the future. Last, the present work is limited to static loading (pullout in test block) without considering of other physiological loadings. In actual physiological situations, the screw/bone interface is subjected to complex dynamic multi-directional loading. Our results cannot be used to predict the biomechanical performance of screw fixation under cyclic loading in the long term. Therefore, the possible future work could be to investigate the fatigue properties of pullout strength of the solid vs. cannulated pedicle screws in animal models.

## Conclusions

We conclude that solid screws with retrograde cement pre-filling offer improved initial fixation strength when compared to that of cannulated screws with cement injection through perforation for both conically and cylindrically shape screws. Our results also suggest that the fixation screws can be backed out by 360 degrees for intra-operative adjustment without the loss of fixation strength.

## Competing interests

The authors declare that they have no competing interests.

## Authors' contributions

LHC and CLT participated in the study design, in collecting the data and drafting of the manuscript. DML participated in the Taguchi analysis. PPL and YCL participated in the experimental work. CCN participated in revising critically the manuscript. WJC advised and assisted drafting of the manuscript. All authors read and approved the final manuscript.

## Pre-publication history

The pre-publication history for this paper can be accessed here:

http://www.biomedcentral.com/1471-2474/12/33/prepub
